# Software tools to support title and abstract screening for systematic reviews in healthcare: an evaluation

**DOI:** 10.1186/s12874-020-0897-3

**Published:** 2020-01-13

**Authors:** Hannah Harrison, Simon J. Griffin, Isla Kuhn, Juliet A. Usher-Smith

**Affiliations:** 1 The Primary Care Unit, Department of Public Health and Primary Care, University of Cambridge, Cambridge, UK; 2 MRC Epidemiology Unit, Institute of Metabolic Science, Cambridge, UK; 3 University of Cambridge Medical Library, University of Cambridge School of Clinical Medicine, Cambridge, UK

**Keywords:** Title and abstract, Screening, Software tools, Feature analysis, Systematic reviews

## Abstract

**Background:**

Systematic reviews are vital to the pursuit of evidence-based medicine within healthcare. Screening titles and abstracts (T&Ab) for inclusion in a systematic review is an intensive, and often collaborative, step. The use of appropriate tools is therefore important. In this study, we identified and evaluated the usability of software tools that support T&Ab screening for systematic reviews within healthcare research.

**Methods:**

We identified software tools using three search methods: a web-based search; a search of the online “systematic review toolbox”; and screening of references in existing literature. We included tools that were accessible and available for testing at the time of the study (December 2018), do not require specific computing infrastructure and provide basic screening functionality for systematic reviews. Key properties of each software tool were identified using a feature analysis adapted for this purpose. This analysis included a weighting developed by a group of medical researchers, therefore prioritising the most relevant features. The highest scoring tools from the feature analysis were then included in a user survey, in which we further investigated the suitability of the tools for supporting T&Ab screening amongst systematic reviewers working in medical research.

**Results:**

Fifteen tools met our inclusion criteria. They vary significantly in relation to cost, scope and intended user community. Six of the identified tools (Abstrackr, Colandr, Covidence, DRAGON, EPPI-Reviewer and Rayyan) scored higher than 75% in the feature analysis and were included in the user survey. Of these, Covidence and Rayyan were the most popular with the survey respondents. Their usability scored highly across a range of metrics, with all surveyed researchers (*n* = 6) stating that they would be likely (or very likely) to use these tools in the future.

**Conclusions:**

Based on this study, we would recommend Covidence and Rayyan to systematic reviewers looking for suitable and easy to use tools to support T&Ab screening within healthcare research. These two tools consistently demonstrated good alignment with user requirements. We acknowledge, however, the role of some of the other tools we considered in providing more specialist features that may be of great importance to many researchers.

## Background

Since the 1980‘s the field of research synthesis has grown exponentially. As the number of primary research papers increases, so does the need for secondary research that consolidates and summarises their findings. Systematic reviews are a form of research synthesis that use systematic methods to find, critically analyse and collate the results of existing studies. In healthcare, systematic reviews are vital to the pursuit of evidence-based medicine; they identify gaps in knowledge and agreement between different studies and provide the evidence required to move confidently from interventions to policy [1, 2]. A report published in 2007 identified 300 systematic reviews indexed in Medline in 1 month [3]. In 2017, 11,000 systematic reviews were registered with PROSPERO [4].

Systematic reviews necessitate screening large numbers of articles to ascertain whether they meet specified inclusion criteria. The first round of screening, typically title and abstract (T&Ab) screening, can be time-consuming. To divide the workload and enable all of the articles to be screened more than once - in accordance with best practice guidelines - this stage of the review is often shared between several collaborators [5, 6]. In response to the growing need for support for T&Ab screening, a large number of software tools have been developed to facilitate this stage of the systematic review process. These tools are a mix of commercial and academic projects, which vary greatly in style, scope and cost. The selection of the most appropriate tool to support a review project or research collaboration will depend on the specific skill set and processes of the local research environment. Previous studies have only reviewed tools that support the entire systematic review process [7, 8], and in each case have largely focussed on tools targeted at specific fields of research (such as agriculture or software engineering).

This scoping review aims to identify, describe and evaluate the usability of the available software tools that support the T&Ab screening process for healthcare research to enable researchers to select the most appropriate for their work. We develop a feature analysis framework to compare software applications for T&Ab screening, using input from researchers to determine the areas of most importance. Additionally, the user experience of several tools is investigated by a survey of several researchers.

This work may be of particular interest to researchers new to systematic reviews, looking to change their approach to screening or those in the position of selecting an appropriate tool for a collaboration.

## Methods

The study had four stages to identify and evaluate the suitability of currently available software tools to support T&Ab screening. The stages are: a search for relevant tools, screening for suitability, a feature analysis and a user survey.

### Search strategy

We developed a search strategy to identify software relevant to this study. Firstly, a web search was conducted. Using a private browser, the first five pages of a google search - “systematic review screening software” - were searched for links or references to relevant software tools. Secondly, we searched the systematic review toolbox, which contains a list of 157 software tools [9]. We obtained a list of suitable tools by using the filter “study selection”. Finally, we included software tools mentioned in two previous reviews of tools to support systematic reviews [7, 8]. The search was carried out in December 2018.

### The identification of tools for inclusion

A list of five criteria for inclusion was developed by three of the authors (HH, JUS and SG); the latter criteria were applied only if the former were met. One researcher (HH) applied the criteria to all the software tools identified by the search. The inclusion criteria, in order of application, are listed below.

1. The software is currently accessible
  i. The website hosting this software tool must currently exist
  ii. It is possible either to access this tool online or download and install the tool
2. It was possible to test the software for free
  i. This requires that either the tool is free to use or there is a free trial available
  ii. Where a free trial was not automatically available, then the company or organization hosting the application was contacted to request one
3. The software has reasonable system requirements
  i. The user is not required to provide specific computing infrastructure (such as an SQL server) in order to use the software
4. Provide basic screening functionality for SRs
  i. The tool can be used to screen references (at least by title and abstract). This requires that there is additional functionality above what is provided by a reference manager
5. The software is working (it is possible to carry out a test project)
  i. The user must be able to carry out basic tasks (such as importing references) with the tool

### Feature analysis

Conducting a feature analysis of a collection of software applications with similar applications is a well-recognised method in software engineering [10, 11]. This involves developing a list of relevant features that a software tool developed for a specific purpose, such as T&Ab screening, might be expected to possess. Each feature is then assessed, for each tool being considered, to generate a score. Analysis of the individual features also provides the evaluator with an insight into the strengths and weaknesses of the individual software tools and the overall group.

As part of the DESMET method (a methodology for evaluating software engineering methods and tools) [12], guidelines for conducting feature analyses of software applications were published by Kitchenham and colleagues in 1993 [13, 14]. The feature analysis developed in this study uses the “screening mode design” described in these guidelines. A list of relevant features was devised by one researcher (HH), in part drawing on previous feature analyses of software tools for systematic reviews [8], as well as consulting with medical researchers involved in systematic reviews. Five researchers participated in a discussion group during which a list of potential features were presented; the researchers added, removed and revised the list of features until a consensus was reached. The features were grouped into themes, such as “Economic” or “Process Management”, providing an easy way to identify areas of strength and weakness in each tool. A single evaluator (HH) devised assessment criteria - which can be found in the supplementary materials (Additional file 1). These were reviewed by a second researcher prior to the collection of information and feature scoring for each tool. Each software tool was evaluated using test projects set up for this purpose. For each feature, software tools were given a score of “0” if the feature did not exist, “1” if the feature existed and “2” if the feature was well implemented.

In order to make the overall score for the feature analysis more reflective of the needs of systematic reviewers in healthcare, a weighting for the features was devised collaboratively by a group of medical researchers. A similar weighting has previously been developed for the assessment of systematic review support tools within software engineering [8]. Two researchers with experience of systematic reviews were interviewed and a further five participated in a discussion group. The researchers were asked to rate features as “Mandatory” (M), “Highly Desirable” (HD), “Desirable” (D), “Nice to Have” (N) or Irrelevant (I). The final rating for each feature was determined by taking the consensus view of the seven researchers. To achieve an overall score for each software tool, the feature scores (0–2) were multiplied by the relevant weighting (0–4, irrelevant-mandatory). The raw score for each tool, achieved by summing the weighted score of each feature, was converted to a percentage of the total possible score. This is necessary, as some features (such as “easy installation”) were not applicable to every software tool evaluated. Radial diagrams, which plot the results of the weighted feature analysis by theme, were plotted for the six best performing tools.

### User survey

We developed a user survey to investigate the opinions of medical researchers involved with systematic reviews on the suitability of the tools. The best performing tools, those scoring higher than 75% in the feature analysis, were included in the user survey. Potential survey participants were recruited using a snowballing approach, from which we were able to select a range of experience levels and career stages. Eight researchers were approached and six agreed to take part in the user survey.

A standard form was developed to record the responses of six researchers to the user survey; this can be found in the supplementary materials (Additional file 2). All the survey respondents had some experience of working with systematic reviews and they encompassed a range of experience levels and career stages. The researchers were asked to provide some information about themselves, their research experience and their attitudes towards software tools for T&Ab screening. They were then asked to run a trial project on a selection of the software tools and report on their experience. For each tool, the researchers were asked to indicate how straightforward a series of seven actions were to complete. The actions were:

1. Creating an account
2. Creating a systematic review project
3. Importing references
4. Inviting collaborators to join the project
5. Carrying out T&Ab screening on the references
6. Exporting the screened references
7. Finding and using the help section

The ease of completing each action was ranked on a scale of 1 to 5 (where 1 is very difficult and 5 is very easy). For each tool, we calculated the “action score”, which is the average score given by the respondent over the seven actions. Radial diagrams were plotted to show the performance of each tool for each of the seven actions. Additionally, an overall score was given by each researcher to each tool on a scale of 1 to 5 (where 1 is very bad and 5 is very good). Additionally, the researchers compared the tools to using a spreadsheet and indicated if they would be likely to use the tool themselves or recommend it to a colleague.

The survey respondents also provided free text comments on the strengths, weaknesses and their general impression of each tool. Issues or topics mentioned by more than one respondent were identified.

## Results

### Identification of software tools

**Fig. 1:**
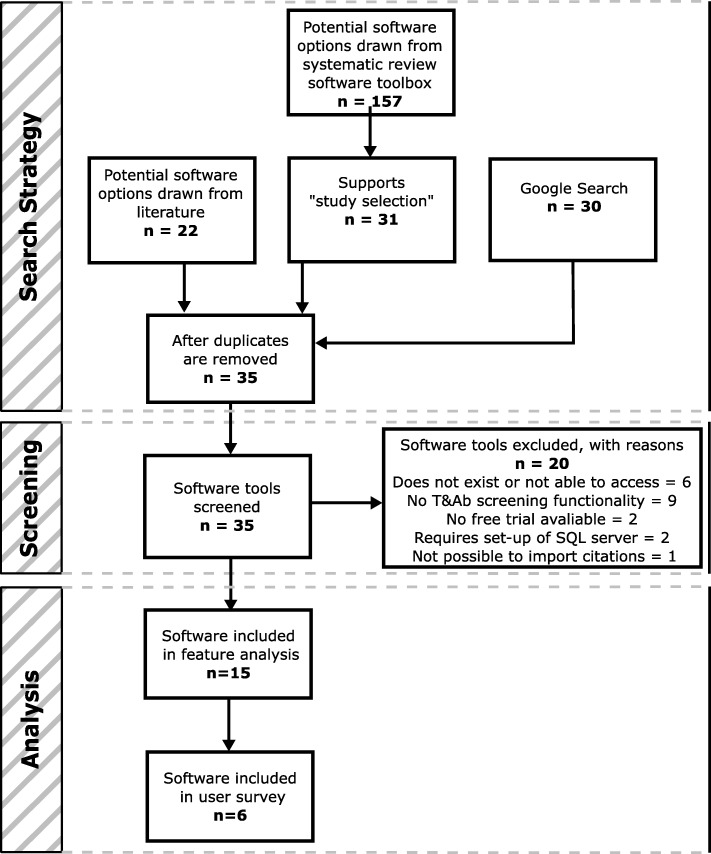
Software tool flow diagram

After deduplication, the search strategy identified 35 software tools that had been characterised by others as supporting the screening phase of systematic reviews. A full list of these software tools, as well as information on where they can be found, is given in the supplementary materials (Additional file 3). Of these, 20 were excluded in the screening process by one reviewer (HH). The most common reason for excluding a tool from the study was that it did not have T&Ab screening functionality, for example the tool JBI SUMARI only supports full text screening and RevMan5 does not provide any screening functionality beyond what is offered by a reference manager. Six software tools were excluded because it was not possible to access them; in most cases because the website hosting the tool is no longer supported (for example GAPScreener). For two tools, DistillerSR or EROS, it was not possible to obtain a free trial, so these were not investigated further. Additionally, SluRp and SLR-Tool were excluded as they required the setup of an SQL server. Finally, although SESRA fulfilled all the other criteria, it was not possible to upload citations to this tool and carry out a test project, so this tool was also excluded. After screening 15 software tools were included in this study (Fig. 1). The included tools ranged from those providing a basic system exclusively for T&Ab screening (for example Abstrackr) to platforms able to offer support for several stages of the systematic review process (for example EPPI-reviewer).

### Feature analysis

The features of the software tools assessed in this study and their weighting are listed in Table 1, they are split into seven themes for the purpose of analysis.

**Table 1:** Overview of feature analysis

Themes	Features	Code	Weighting^a^
Economic	The tool does not require financial payment to use.	T1-F1	HD
Ease of Introduction and Setup	The tool has straightforward system requirements	T2-F1	HD
	There is an installation guide (where applicable)	T2-F2	D
	There is a tutorial/help section	T2-F3	D
	The software does not require user to code	T2-F4	HD
	There is an app for mobile/tablet	T2-F5	D
Systematic Review Support	Supports deduplication	T3-F1	D
	Supports title and abstract screening	T3-F2	–
	Supports full text screening	T3-F3	D
	Supports data extraction	T3-F4	N
	Supports other stages of the review	T3-F5	N
Process Management	Support for multiple users	T4-F1	M
	Support for multiple projects	T4-F2	D
	Choice of single or double screen before progression	T4-F3	HD
	Work Allocation	T4-F4	HD
	Management of roles	T4-F5	D
Reference Management	Import of References	T5-F1	–
	Export of References	T5-F2	M
	Export of Decisions	T5-F3	M
	Import of .pdfs	T5-F4	D
Workflow	The tool is flexible to varying workflow	T6-F1	HD
	Short User Set-up (before screening can begin)	T6-F2	D
	Progress is monitored and fed back to user	T6-F3	HD
Screening Features	Include/Exclude Option	T7-F1	–
	Key word highlighting (or similar)	T7-F2	D
	Can filter citations by category	T7-F3	D
	Can search citations (i.e. search engine)	T7-F4	D
	Further categorize/label references	T7-F5	HD
	Blind screeners to decisions of others.	T7-F6	HD
	Conflict Resolutions	T7-F7	HD
	Citation classification/ranking tool (clustering/ML)	T7-F8	N
Security	Insecure website	T8-F1	HD

Features without a weighting are covered by the inclusion criteria and are found in all the included software tools

^a^*Abbreviations*: *M* Mandatory, *HD* Highly Desirable, *D* Desirable, *N* Nice to Have, *I* Irrelevant

**Fig. 2:**
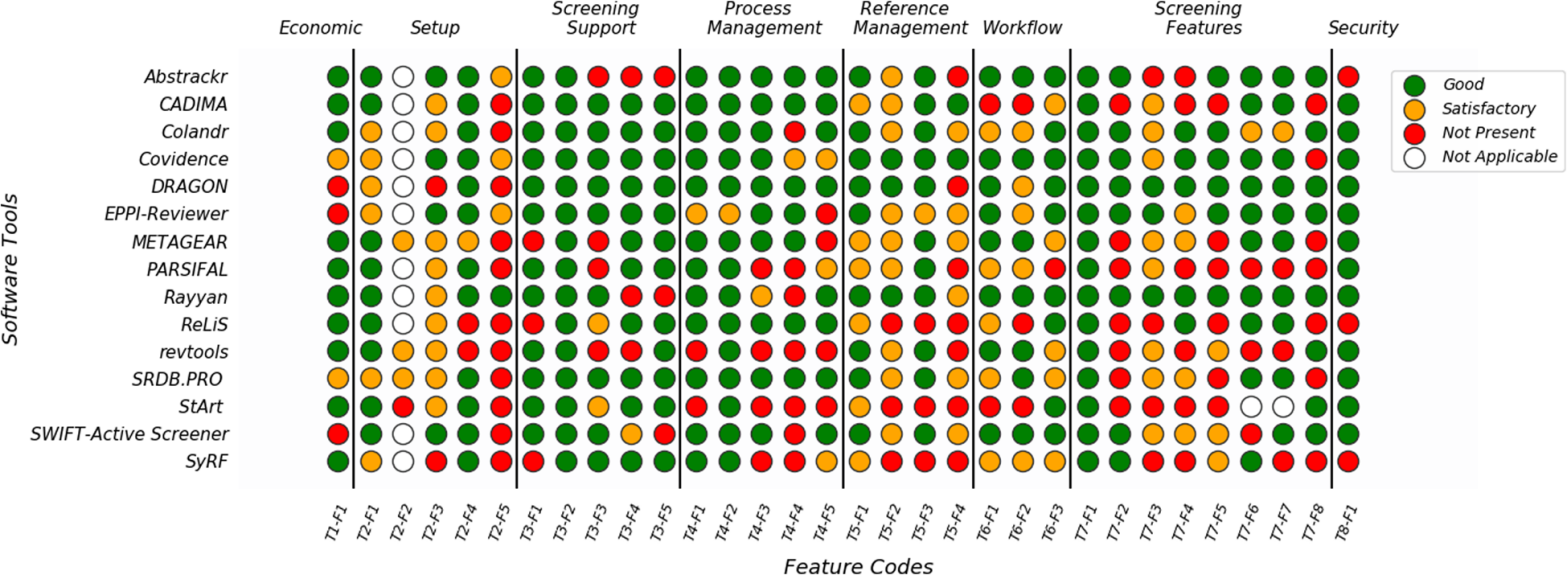
Traffic light diagram of software features. Red indicates that the feature is not present, orange that it has been implemented, and green that it has been implemented well. Feature codes can be seen in Table 1

The features of each software tool are summarised in the traffic light diagram (Fig. 2). Two of the features - supports title and abstract screening (T3-F2) and has an include and exclude option (T7-F1) - are implemented well in all the tools. In both cases, these features are ensured by the inclusion criteria and do not contribute to the score assigned to the software tools in the subsequent analysis.

A large amount of variation was seen between the 15 software tools. For the features grouped under the theme of “screening features”, DRAGON and Rayyan had all these features rated as well implemented (green), however, PARSIFAL only had two of them implemented (and only one implemented well).

Some of the features considered were very common and were implemented in the majority of tools. For example, all the tools considered supported multiple projects (T4-F2) and all but one implemented this well. The only tool that did not implement this well was EPPI-reviewer, as users were required to pay more for additional projects. More generally, most of the tools had the features grouped under the theme “screening support” (T3) well implemented. This reflects that most of the tools considered support multiple stages of systematic reviews.

There are, however, features that were not implemented for many of the 15 tools evaluated. These include providing a mobile (or tablet) application (T2-F5) - which was only well implemented in one tool (Rayyan) - and supporting the import of .pdf files for full text screening (T5-F4) - which was only implemented well in two tools (CADIMA and Covidence).

**Fig. 3:**
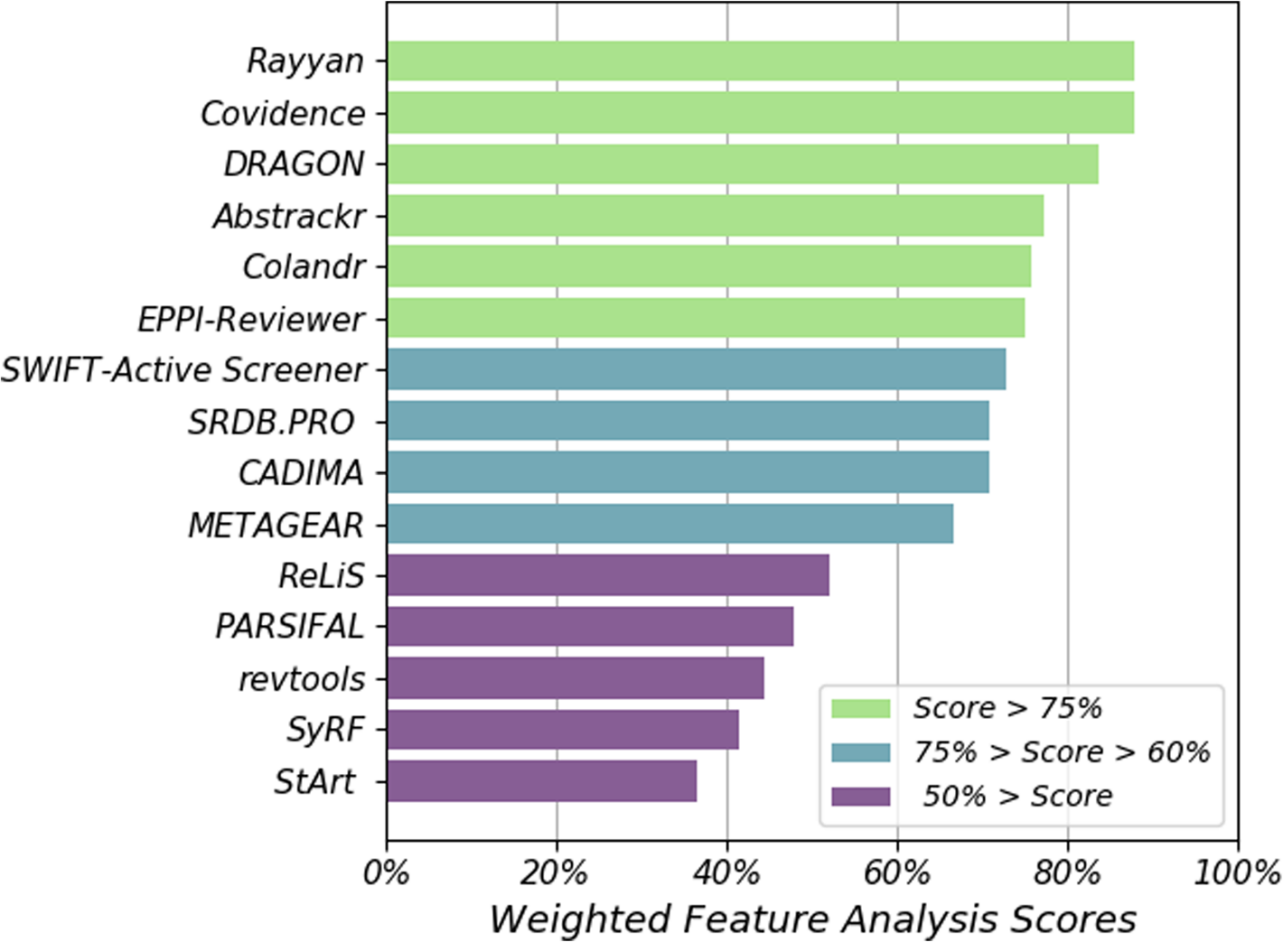
Scores from the weighted feature analysis, with the software tools ranked from lowest to highest. The scores are given as a percentage of the total possible score

The 15 software tools are ranked from highest to lowest in Fig. 3 according to the summary score based on the feature information (Fig. 2) and the weighting (Table 1).

The scores for the weighted feature analysis, as shown in Fig. 3, ranged from 88% (Rayyan and Covidence) to 36% (StArt). All of the tools designed specifically for non-medical researchers - including CADIMA (agriculture) and PARSIFAL (software engineering) – scored less than 75% in the feature analysis. Additionally, both of the tools that require the user to use the R programming language, METAGEAR and revtools, scored less than 75%.

Radial diagrams, showing the scores of the six best performing tools across the eight themes (as identified in Table 1) can be found in the (Additional file 4: Figure S1).

### User survey

Six healthcare researchers ran a test project in each of the six highest scoring software tools from the feature analysis. A brief summary of the experience and attitude of the survey respondents towards software tools for T&Ab screening is given in Table 2.

**Table 2:** Description of survey respondents

Categories	Respondent characteristic	Number (*n* = 6)	Percentage
Research Position	*Medical Student*	1	16.7%
	*PhD Student*	1	16.7%
	*Postdoctoral Researcher*	3	50.0%
	*Medical Librarian*	1	16.7%
Systematic Review Experience	*Carried out T&Ab Screening*	6	100%
	*Managed T&Ab Screening*	4	66.7%
	*Led a Systematic Review*	4	66.7%
Number of Systematic Review Projects in the Last Year	*0 to 2*	1	16.7%
	*3 to 5*	2	33.3%
	*6 to 10*	2	33.3%
	*More than 10*	1	16.7%
Current T&Ab Screening System	*Reference Manager*	3	50.0%
	*Spreadsheet*	1	16.7%
	*Software Package*	2	33.3%
Previous Software experience	*Used software previously*	6	100.0%
	*Rayyan*	5	83.3%
	*Covidence*	2	33.3%
Preferences for T&Ab screening	*Would prefer to use a software tool*	6	100%
	*Believe a software tool would be well received by collaborators*	6	100%

Summary of the experiences of the survey respondents with systematic reviews and their attitudes towards software to support T&Ab screening

All six respondents completed the survey; however, one respondent was unable to use EPPI-reviewer due to problems installing the Silverlight application. Therefore, only five responses were available for the analysis of this tool.

**Fig. 4:**
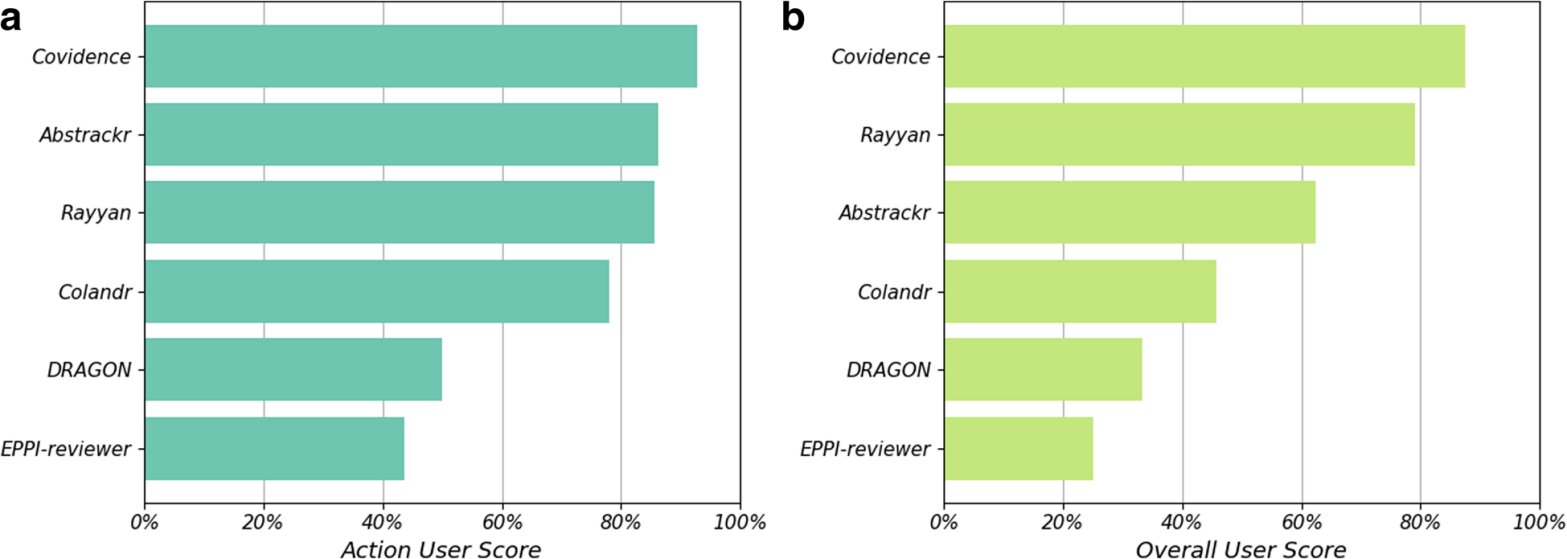
The performance of the six software tools evaluated in the user survey are compared using **a** the action score (the average score over the seven key actions) and **b** the overall score (a single score provided for each tool indicating overall experience)

The action and overall scores obtained for each tool, using the survey responses, are given in Fig. 4a and b respectively; both are given as percentages of the highest possible score.

For both measures shown in Fig. 4, Covidence received the highest score (93 and 88% respectively). Rayyan and Abstrackr both scored 86% on the action score, however, for the overall score Rayyan performed significantly better than Abstrackr (79 and 62% respectively). Colandr, DRAGON and EPPI-reviewer consistently performed worse than the other three tools.

Additional file 5: Figure S2 plots the breakdown of the average scores for each of the seven actions for each of the seven actions. Whilst in most categories Rayyan and Abstrackr had similar performance (Additional file 5: Figure S2(f) and (a)), Rayyan performed significantly worse for the action “find and use the help section”, indicating that several respondents had problems with this task. However, the respondents’ free text comments suggest that this was not a significant issue. Comments including - “I wouldn’t have looked for one <a help section>” and “it’s fairly self-explanatory” – suggest that for this software tool the users did not find the lack of a good quality help section particularly important when giving their overall scores. Abstrackr, although it performed well in each of the seven action categories, did not perform as well in the overall scores. Free text comments from the respondents, describe Abstrackr as “basic”, “informal” and not having “as much functionality as other tools”, which may explain the lower overall rating.

Table 3 shows the common themes that emerged from the comments made about each tool with indicative quotations. All of the survey respondents identified Rayyan as a tool that was simple or easy to use. This provides good supporting evidence for the high overall score this tool received. In contrast, all of the survey respondents indicated that DRAGON was hard to setup compared to other tools; five (out of six) respondents stated that they would require training in order to use DRAGON effectively. Similarly, five of the respondents indicated that they found EPPI-reviewer difficult to use (note that only five respondents were able to use EPPI-reviewer) and that they would require training to use it effectively.

**Table 3:** Identified strengths and weaknesses of the software

	Strengths		Weaknesses	
	Themes	Supporting Quotations	Themes	Supporting Quotations
Rayyan	*Simple and easy to use. (6)*	“So easy to use..” “Very simple to import and export.”	Too much content on the side panel. (2)	“I don’t think the sections of the left side of the screen are helpful” “<it suggests> keywords to include/exclude - it means you have to start by deleting theirs”
DRAGON	Flexible/provides many options. (3)	“..there might be many things you could do with DRAGON...” “..lots of flexibility in the set-up…”	*Hard to setup (compared to other tools). (6)*	“Quite complicate initially to set up..” “Not clear how to set up and use.”
Abstrackr	*Simple option for “basic” screening. (5)*	“..simple screening method…” “Easy to set-up and do basic screening.”	Poor quality user interface. (3)	“..it felt a bit clunky..” “Not a very professional website…”
	Good for collaborating. (2)	“...flexible in terms of team working” “Good options for collaborative projects”	Format of exported citations. (2)	“Exporting not as clear format as Covidence or Rayyan.” “..0 or − 1 as identifiers of exclude are ambiguous..”
EPPI-reviewer	Could be useful in large/ complex projects with multiple stages. (3)	“..it could be helpful with all stages of the review.” “good coding elements … < for>..a very large review”	*Complex, difficult (not intuitive) to use. (5)*	“Makes the screening very cumbersome.” “Very bad layout that is not self-explanatory.”
			Difficulties getting started. (2^a^)	“Difficult to access and start using…” “Had to install software and use internet explorer.”
			Instructions/help section. (2)	“..needed to watch a slow video for instructions.” “Could not find help section/tutorial.”
Covidence	Easy to use, good user interface. (3)	“Simple user interface…” “.. - very clear and simple, not too much information on the page.”	Issues with help section. (2)	“..no clear help function “…videos in help section didn’t have subtitles …difficult to use if no volume/in office”
	Able to export into many formats. (2)	“..able to export citations and decisions into Excel and < reference managers>“ “...ability to export as .ris or .csv …”		
	Supports PRISMA flow diagram (and other extra features). (3)	“...ability to generate PRIMSA flowchart…” “Some of the extra features are nice (e.g. …generate a PRISMA diagram)...”		
Colandr	Easy to use/good interface. (3)	“…aesthetically pleasing <the interface>… and simple to use.” “Simple user interface…”	Required to provide exclusion reason at title and abstract stage. (2)	“…you have to give a reason for exclusion at title and abstract stage…” “..having to include a reason for all exclusions”
	Easy to import citations. (2)	“Simple to import.” “...easy to import…”	*Slow processing of decisions (especially excluded citations). (4)*	“…a little slow to respond…” “...excluded citations not disappearing…”

Identified themes from the free text comments by survey respondents regarding the strengths and weaknesses of each tool for T&Ab screening. In each case the number of respondents who identified the theme is indicated (themes identified by four or more respondents are in bold). Indicative quotations are provided for each theme

^a^Only five of the six respondents were able to use – and give responses for – EPPI-reviewer

Four respondents mentioned that Colandr was slow to process decisions and that the excluded decisions did not disappear immediately. As saving time was identified (by three researchers) as a motivator for their interest in software for T&Ab screening, a software tool with a slow response is unlikely to score highly.

All the respondents indicated that Abstrackr, Rayyan and Covidence performed well and made screening easier (or much easier) than using a spreadsheet. The overall view of Colandr was that the experience of using it for T&Ab screening is comparable to using a spreadsheet. However, there was variation in views with some respondents positive about its performance whilst others did not feel that it was a useful tool. Similarly, whilst overall the respondents thought that using EPPI-reviewer or DRAGON would be more difficult than using a spreadsheet, the extent of this varied between the respondents. Several of the respondents that scored EPPI-reviewer or DRAGON highly mentioned the potential to carry out complex tasks, using some of the additional features and flexibility, with these tools. Other respondents, however, focussed on the difficulties of getting started and navigating a more difficult user interface.

Finally, responses to the question ‘how likely are you to use the tool in future?’ varied. While all of the respondents said they would be likely to use Rayyan, there was less consensus about using Covidence. Several free text comments from the respondents mentioned the cost of using Covidence – “so expensive”, “I wouldn’t be able to choose to use this tool, since there is a cost” – as a reason why they would not typically use this tool for systematic reviews.

## Discussion

### Key findings

We have identified a large number of software tools that support T&Ab screening for systematic reviews within healthcare research. Where possible we have tested the software and identified relevant features. The six highest scoring tools were trialled by a group of six healthcare researchers with experience of systematic reviews. Out of all the software tools considered, Covidence and Rayyan emerged as the most suitable tools to support T&Ab screening for systematic reviews in both the feature analysis and the user survey.

### Findings in the context of other research

Other reviews of software tools to support systematic reviews have reported different findings. An analysis by Kohl et al. [7] of software tools to support the conduct and reporting of systematic reviews found CADIMA to be the most useful open access tool. A feature analysis of tools to support systematic reviews in software engineering carried out by Marshall et al. [8] recommended SLuRp as the highest scoring tool.

CADIMA and SluRp were amongst the software tools identified during the search for this study; both were eliminated during the early stages of the review process. SluRp was not considered appropriate for this study, as its use requires the setup of an SQL server. CADIMA was evaluated in the feature analysis, but did not score highly enough to be included in the user survey. In particular, CADIMA did not have many of the screening features that were considered very important by the systematic reviewers we consulted, such as, allowing users to add additional labels and categories other than simply “include” and “exclude”. The disparities in outcome not only reflect the different evaluation frameworks used, but also the diverse priorities of each academic community of systematic reviewers.

The studies by Kohl [7] and Marshall [8] differ significantly from this one in scope (T&Ab screening) and target audience (healthcare researchers). Both Kohl and Marshall only consider tools that support the entire systematic review process. The study by Kohl focuses on how software tools can support systematic reviewers to achieve best practice and transparency in reporting. The aim of the report is to demonstrate the benefits of the newly developed CADIMA for systematic reviewers of genetically modified crops. The framework used to evaluate the tools is based on the features within the CADIMA tool.

Marshall and colleagues carried out an independent evaluation of four “whole process” tools for systematic reviews, used in the software engineering community. In the analysis, a single feature addresses “study selection and validation”. Therefore, neither of these studies provides a thorough evaluation of tools to support T&Ab screening. Several tools evaluated in this study, including Abstrackr, are not considered by either Kohl or Marshall.

### Strengths and limitations

The development of a new feature analysis strategy allowed for a transparent evaluation of the available software tools. We were able to provide detailed information about the features available within the 15 tools evaluated (Fig. 2) and calculate an overall score that reflects the priorities of the user community (Fig. 3). Whilst we have drawn heavily on existing methods, including studies of systematic review tools in the software engineering community, this framework was specifically designed for assessing screening tools. To the best of our knowledge, this type of method has not been applied within the medical research community previously.

In combining the feature analysis with the user survey, this study provides a comprehensive evaluation of T&Ab screening tools. The feature analysis syntheses a large amount of detail that is not necessarily relevant to all of our survey respondents. On the other hand, the user survey reveals how well the tool is designed and how cohesive the experience of using it is, which is not measured by the feature analysis. The agreement seen between these two methods, with Rayyan and Covidence performing best in both, suggests that there is some correlation between these two aspects.

Collecting both quantitative and qualitative data in the user survey improved our understanding of the usability of the screening tools. The quantitative approach allows us to rank the tools in the analysis and directly compare measures of performance in a variety of areas. Collecting qualitative data as well, such as the free text about the strengths and weaknesses of each tool, makes it possible to investigate in more depth the reasons for the quantitative scores. This highlights issues of importance to the user community and is a useful source of information for both the users and developers of these tools.

There are several limitations of this study, which should be considered when interpreting its findings. This study only considers the features offered by each tool and the user experience they provide. We have not, when carrying out this work, assessed other measures of performance that may be of interest to potential users of these tools. These include, but are not limited to, the following: the extent to which the tool, or its features, supports users to identify a high proportion of eligible studies; the extent to which the design of the tool supports reviewers to accurately record their decision and limits accidental misclassification; and the reliability with which work done by a reviewer is recorded. Readers who are considering using the tools discussed in this study should consider these properties in addition to considering the user experience.

The tools identified by the search displayed considerable heterogeneity, which makes drawing comparisons between them more difficult. Additionally, in this study, we have chosen to consider the T&Ab screening stage in isolation. While this means we were able to compare a wide range of tools that offer that function, our findings do not consider the potential advantages or disadvantages of using a simple tool just for T&Ab screening or a platform that supports multiple stages of the systematic review process.

It was not possible within the scope of this project to test all of the software tools identified. Tools that did not provide a free trial - either automatically or when requested – were not evaluated. This excluded several well-known tools, such as, DistillerSR and EROS which have been reported on elsewhere [7]. Additionally, this study did not consider tools which required the user to setup their own MySQL server (or similar) to be appropriate for general use. Therefore, tools including SLR-TOOL and SLuRp have not been evaluated. Discussion with systematic reviewers (both when developing the weighting scheme and carrying out the user survey) revealed little appetite for lengthy or complex setup of software tools unless significant savings in time or resources could be made.

This study also relied heavily on one researcher (HH), who carried out all the screening as well as designing and implementing the feature analysis. The dependence on the subjective opinion of one researcher could have biased the findings. This was mitigated in the feature analysis by consulting other researchers. The contributions of several researchers were included when developing the list of features and a discussion group was used to develop the weighting score. This helped to broaden the perspective of the feature analysis, in order to be more representative of the medical research community.

The number of participants in the user survey was small (*n* = 6), therefore caution is required when interpreting the findings. There is also the potential for respondent bias amongst the researchers who completed the user survey. The six respondents were all drawn from the same research community and all but one work in the same department. Additionally, all of the respondents had some previous experience with either Rayyan or Covidence and this may have resulted in a bias in favour of these tools. Some of the free text comments support this hypothesis, with Rayyan in particular described as “familiar” by two respondents. Furthermore, in order to simulate a “real-world” user experience when testing the six tools included in the survey, the participating researchers were not given any external guidance on how to use each of the tool. This would have made the experience of using a familiar tool straightforward when compared to an unfamiliar tool, particularly when considering the more complex tools (such as EPPI-reviewer or DRAGON).

It was noted by the authors, when carrying out the review that this area is subject to relatively fast-paced changes. Six of the tools identified by the search were excluded because they no longer exist, or are no longer accessible (see Fig. 1). During the timeframe of this study (since November 2018), new upgrades have been made to CADIMA (included in this study) and a web-based version of EPPI reviewer (currently a beta-version, more updates expected until the end of 2019, not included in this study) has been launched. DRAGON has only recently been developed and is not yet widely available; it is currently being rebranded as litstream and further changes are expected in the coming months. While the diverse range of tools available already (some of excellent quality) is encouraging, systematic reviewers will be pleased to know that development and innovation are ongoing in this area.

## Conclusions

We identified 15 tools that can be used to support T&Ab screening for systematic reviews in medical research. Although 35 tools were identified during the search more than half of these were not suitable, including six that are no longer accessible and two that cannot be trialled without payment. We developed a feature analysis framework, the results of which showed that there is a large amount of variation in the properties and the quality of these tools. In the user survey, which looked at the user experience of only the six highest performing tools, a range of quality was also found.

The results of this study suggest that Covidence and Rayyan provide the best user experience for systematic reviewers carrying out T&Ab screening. These two tools consistently performed well for the range of measures we used. We acknowledge, however, the role of some of the other tools we considered in providing more specialist features that may be of great importance to many researchers.

## Supplementary information

**Additional file 1.** Feature analysis assessment.

**Additional file 2.** Questionnaire template.

**Additional file 3.** Complete list of software tools (with urls).

**Additional file 4: Figure S1.** Radial diagrams of six highest performing tools in the feature analysis. The performance of the six highest scoring software tools in the feature analysis by theme. The software tools in each plot are (a) Abstrackr, (b) Colandr, (c) Covidence, (d) DRAGON, (e) EPPI-Reviewer and (f) Rayyan. The themes are: (T1) Economic, (T2) Ease of Introduction and Setup, (T3) Systematic Review Support, (T4) Process Management, (T5) Reference Management, (T6) Workflow, (T7) Screening Features and (T8) Security. The features included in each theme can be found in Table 1.

**Additional file 5:** **Figure S2.** Radial Diagrams showing performance of the six highest performing tools in the user survey. The performance of the software tools in the user survey, considering the average score for each of the seven actions. The software tools in each plot are (a) Abstrackr, (b) Colandr, (c) Covidence, (d) DRAGON, (e) EPPI-Reviewer and (f) Rayyan. The actions are: (A1) creating an account, (A2) creating a systematic review project, (A3) importing references, (A4) inviting collaborators to join the project, (A5) carrying out T&Ab screening, (A6) exporting the screened references and (A7) finding and using the help section.

## Data Availability

All quantitative data generated or analysed during this study are included in this published article and its supplementary information files. Free text responses to the user survey are available from the corresponding author on reasonable request.

## Abbreviations

DESMET: A methodology for evaluating software engineering methods and tools. The DESMET project identified nine methods of evaluation and a set of criteria to help with the selection of an appropriate method
PRISMA: The PRISMA statement consists of a checklist and a flow diagram that give a minimum set of items for reporting in systematic reviews and meta-analysis. The PRISMA flow diagram in used in this study to report the number of software tools at each stage of the selection process
T&Ab screening: Title and Abstract screening
