# Supplementary material for: Software tools to support title and abstract screening for systematic reviews in healthcare: an evaluation
Source: BMC Med Res Methodol. 2020 Jan 13;20:7. doi: 10.1186/s12874-020-0897-3 (PMC6958795; doi:10.1186/s12874-020-0897-3)
Supplement: Supplementary file 1 — Additional file 1. Feature analysis assessment. [file 12874_2020_897_MOESM1_ESM.docx]

**Feature Analysis: Discussion and Definitions**

**Economic Feature Set**:

- **F1-SF01**: This tool does not require financial payment to use

**Always Free | Sometimes Free | Never Free**

*Should consider different financial settings. In some cases, it will be possible to use grants to buy licences to software. There will also be opportunities for researchers to use departmental or institutional licences. It may be possible for researchers to use free software on one project (e.g. using Covidence as a Cochrane user) but not for others (e.g. when working with collaborators from another institution).*

**Introduction and Setup Features:**

How easy it is to get the software running. This may depend on the skill sets of the researchers involved in the work.

- **F2-SF01:** The tool has straightforward system requirements

**No restrictions | Some restrictions | Very Limited**

*Can any/all operating systems (i.e. only runs on windows) or web browsers (i.e. not supported on IE) be used?*

- **F2-SF02:** There is an installation guide

**Exists and high quality| Exists but poor quality | Doesn’t exist**

*This is only applicable in scenarios where either the software must be installed on the computer or there is a prerequisite which has to be installed (i.e. Microsoft Silverlight for EPPI reviewer) or both.*

- **F2-SF03:** There is a tutorial and/or user guide

**Exists and high quality| Exists but poor quality | Doesn’t exist**

*Is there information (easily) available explaining how to get started using the software and/or is there a document listing and explaining all of the features? A tutorial could include an “interactive walkthrough”. Obviously, the demand for this is dependent on the ease of use of the software and different working styles, in some cases it might not be required or may only be required for advanced use.*

- **F2-SF04:** The software does not require the user to code

**Users only interact with a GUI| At least one user (e.g. admin) has to code | Everybody has to use programming interface**

*This depends on the skill set of the researchers. Typically, this would involve a small set of custom commands in a high-level language (such as METAGEAR, an R module). May not actually require prior experience, but obviously prior experience would make this easier and less daunting to researchers.*

- **F2-SF05:** There is an app for mobile and/or tablet

**Yes | Partial | No**

*This is critical for “on-the-go” screening. Partial implementation includes tools like Covidence, where the website works well on phone/tablet, but there is no separate mobile app.*

**Systematic Review Support:**

This section looks at the support given to various stages of the systematic review. The software discussed in this review has been selected because it supports T&Ab screening; however, many packages support other stages of the process. It may be desirable for researchers to conduct as much of their systematic review using one software tool – however – with added review stage report many come additional complexity and a lack of flexibility. We acknowledge that the quality of the support for each stage is important, however, this is not a consideration for this feature set.

- **F3-SF01:** Supports Deduplication

**Yes | No**

- **F3-SF02:** Supports T&Ab Screening

**Yes | No**

*Inclusion criteria, mandatory for the purpose of this study.*

- **F3-SF03:** Supports full text screening

**Yes | No**

- **F3-SF04:** Supports data extraction

**Yes | No**

- **F3-SF05:** Supports other stages of the review

**Yes | No**

**Process Management Function Set:**

- **F4-SF01:** Support for multiple users

**Yes| Yes, but costs more | No**

*The software provides a means to collaborate with the other screeners – sharing references, tracking the decisions of multiple users.*

- **F4-SF02:** Support for multiple projects

**Yes| Yes, but costs more | No**

*Note that if the software does not provide multi-stage support or easy allocation to single/double screening, multiple projects could be used to host different chunks of citations (i.e. lead researcher may use for allocation/structure).*

- **F4-SF03:** Choice of single or double screening (before progression)

**Yes| In some cases | No**

*Provides the option of single or double screening for each project/stage. For example, software that forces users to screen everything twice before “progression”, receives “0” for this feature.*

- **F4-SF04:** Work Allocation (how dynamic)

**Yes, users have custom allocation| Users have some control over allocation| No**

*How important to researchers is it that the software will provide dynamic work allocation? This could be specific allocation of a certain number of references to individual screeners or random allocation where users specify a percentage must be double screened (CADIMA beta). Ability to control first/second reviewer.*

- **F4-SF05:** Management of roles (admin rights, who controls who sees what?)

**Yes| Yes, but costs more | No**

*This could include: only lead researcher/admin can see conflicts (DRAGON), change the blind mode (Rayyan), or invite other users. Alternatively, only people with certain permissions can change the properties of a screening stage.*

**Reference Management:**

- **F5-SF01:** Import of references

**Yes, supports .RIS (or similar) and spreadsheets| Yes, at least one format supported| No**

***This is a mandatory feature.***

*It is necessary that users are able to upload files containing large numbers of citations (as opposed to manual input of references only). Ideally, the tool will support more than one format, including spreadsheets.*

- **F5-SF02:** Export of references

**Yes, can be filtered | Yes, but no filtering| No**

*The users will also need to be able to export the citations again at the end (presumably filtered or labelled with the decision). Ideally, they would be able to select an appropriate format and no information would have been lost in the process. If the software continues to later SR stages, but does not offer export at the end of T&Ab screening, it would score “0” for this feature.*

- **F5-SF03:** Export of Decisions

**Yes | No**

*In addition to exporting the references, they should also be able to export (either with the references or separately) their decisions.*

- **F5-SF04:** Import of .pdfs (full texts)

**Yes, supports batch upload| Yes, but single upload at a time only| No**

*If the tool also supports full text screening, are users able to import the full texts?*

**Workflow (ease of use):**

- **F6-SF01:** Flexible to varying workflow

**Very flexible| Some flexibility| No flexibility**

*Can the number of screening stages and their properties (e.g. number of screeners) be varied?*

- **F6-SF02:** Short user setup

**Can begin screening without completing other stages| Some additional (planning?) steps are required| Large amount of user work to get to screening stage (design/protocol etc.)**

*Can screening be begun quickly, without a long setup process? How much setup is acceptable in exchange for more features/flexibility?*

- **F6-SF03:** Progress is monitored and fed back to the user

**Yes, overview of whole project| Yes, only your own assignment| No**

*How important to researchers is it that they are given real time feedback. Of their own work allocation? Of the progress of the whole project? Of the progress of other screeners?*

**Screening Features:**

- **F7-SF01:** Include and Exclude option (mandatory!)

**Yes | No**

- **F7-SF02:** Key word highlighting (or similar)

**Yes | No**

*Can keywords be highlighted (potentially using positive/negative colours?) in the titles and abstracts they are screening?*

- **F7-SF03:** Can filter citations by category

**Yes, many options | Yes, some options | No**

*Can uploaded citations be filtered (within the screening paradigm) by various parameters (e.g author, journal, year of publication/keyword present in title or abstract).*

- **F7-SF04:** Search engine, to search citation fields

**Yes, searches all fields including abstract | Yes, searches at least title | No**

*Can users search the citation fields (for specific authors, keywords etc.)*

- **F7-SF05:** Citations can be grouped/labelled (other than I/E)

**Yes | Yes, can add individual comments but not group| No**

*Can the users attach labels (or otherwise group citations), beyond separating into include and exclude categories? Can notes be attached to specific citations?*

- **F7-SF06:** Screeners are blinded to the decisions of others

**Yes | Partially | No**

*Ideally, users are blinded to the decisions of other reviewers when making include/exclude decisions. A review may be partially blinded if the user sees the conflict arise immediately after making a decision (this may encourage users to immediately reverse their choice?).*

- **F7-SF07:** A conflict resolution system handles disagreements between screeners

**Yes, with permissions/allocation | Yes, no restrictions on who can resolve | No**

*Is these are system to manage conflict resolution, and who has control over how/when this is done. Ideally, an admin can assign conflict resolution to one (or more) reviewer(s). Does somebody have to change their original decision explicitly to resolve the conflict (this may fit well with some workflows but not others)?*

- **F7-SF08:** The citations are ranked/clustered by the software (ML tool or otherwise)

**Yes | No**

*Some system/algorithm is contained within the tool that scores the likelihood of the citation being included. In all cases, the tool ranks the citations but does not make any decisions.*

**Security:**

Is security something you think about when using software (particularly online software?). Do you have concerns about security? About the confidentiality of data (user names/passwords/ unpublished research/ownership)? Are your concerns different between commercial/free/open-source software?

- **F8-SF01:** There is a secure connection to the website hosting the tool (or the website hosting the download of a tool)

**Yes | No**

*Users may be uncomfortable using an insecure connection, in particular if they are responsible for sharing an insecure website with colleagues. Additionally, this could be a sign that the tool is not being properly maintained (i.e. no one has updated the security certificate?) and that there would not be any support from the developers.*
