# Supplementary material for: Software tools to support title and abstract screening for systematic reviews in healthcare: an evaluation
Source: BMC Med Res Methodol. 2020 Jan 13;20:7. doi: 10.1186/s12874-020-0897-3 (PMC6958795; doi:10.1186/s12874-020-0897-3)
Supplement: Supplementary file 3 — Additional file 3. Complete list of software tools (with urls). [file 12874_2020_897_MOESM3_ESM.docx]

**List of Software Tools Considered in Study**

Included

| Name | Website |
| --- | --- |
|  |  |
| Abstrackr | [http://abstrackr.cebm.brown.edu](http://abstrackr.cebm.brown.edu/) |
| CADIMA | <https://www.cadima.info/index.php> |
| Colandr | <https://www.colandrapp.com/> |
| Covidence | <https://www.covidence.org/home> |
| DRAGON | [https://icfdragon.com](https://icfdragon.com/) (now <https://www.icf-litstream.com>) |
| EPPI-Reviewer | <http://eppi.ioe.ac.uk/cms/Default.aspx?tabid=2914> |
| METAGEAR package for R | <https://cran.r-project.org/web/packages/metagear/index.html> |
| PARSIFAL | <https://parsif.al/> |
| Rayyan | <https://rayyan.qcri.org/> |
| ReLiS | <http://relis.iro.umontreal.ca/auth.html> |
| revtools | <https://cran.r-project.org/web/packages/revtools/index.html> |
| SRDB.PRO | <https://www.srdb.pro/default> |
| StArt | <http://lapes.dc.ufscar.br/tools/start_tool> |
| SWIFT-Active Screener | https://swift.sciome.com/activescreener |
| SyRF | <http://syrf.org.uk/> |

Excluded

| Name | Website |
| --- | --- |
|  |  |
| DBPedia | <https://wiki.dbpedia.org/> |
| DistillerSR | <https://www.evidencepartners.com/> |
| EROS | <http://www.eros-systematic-review.org/> |
| GAPScreener | ~ |
| HAWC | <https://hawc.readthedocs.io/en/latest/> |
| JBI-SUMARI | <https://www.jbisumari.org/> |
| Lingo 3d | <https://carrotsearch.com/lingo3g/> |
| MeSHSIM | <https://github.com/JingZhou2015/MeSHSim> |
| PEx | **~** |
| Pimiento | [http://erabaki.ehu.es/jjga/pimiento/ (Broken)](http://erabaki.ehu.es/jjga/pimiento/%20(Broken)) |
| REviewER | <https://sites.google.com/site/eseportal/tools/reviewer> |
| ReVis | ~ |
| RevMan 5 | <https://community.cochrane.org/help/tools-and-software/revman-5> |
| RobotAnalyst | <http://nactem.ac.uk/robotanalyst/> |
| SESRA | <http://sesra.net/> |
| SLR.qub | <https://github.com/gmergel/SLR.qub> |
| SLR-Tool | ~ |
| SLuRp | <https://codefeedback.cs.herts.ac.uk/SLuRp/> |
| SWIFT-Reviewer | https://www.sciome.com/swift-review/ |
| Systematic Review Accelerator | <http://crebp-sra.com/#/> |
| SLR Tool (Middlesex) | ~ |
| SLRTOOL | [www.slrtool.org](http://www.slrtool.org) |
