# Supplementary figures and images for: Software tools to support title and abstract screening for systematic reviews in healthcare: an evaluation
Source: BMC Med Res Methodol. 2020 Jan 13;20:7. doi: 10.1186/s12874-020-0897-3 (PMC6958795; doi:10.1186/s12874-020-0897-3)

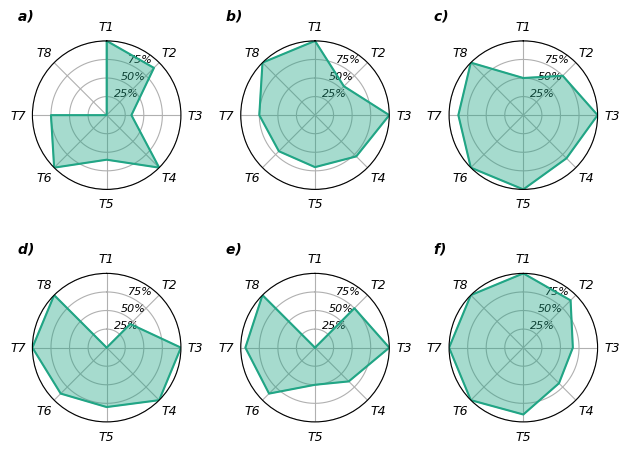

Supplement: Supplementary file 4 — Additional file 4: Figure S1. Radial diagrams of six highest performing tools in the feature analysis. The performance of the six highest scoring software tools in the feature analysis by theme. The software tools in each plot are (a) Abstrackr, (b) Colandr, (c) Covidence, (d) DRAGON, (e) EPPI-Reviewer and (f) Rayyan. The themes are: (T1) Economic, (T2) Ease of Introduction and Setup, (T3) Systematic Review Support, (T4) Process Management, (T5) Reference Management, (T6) Workflow, (T7) Screening Features and (T8) Security. The features included in each theme can be found in Table 1. [file 12874_2020_897_MOESM4_ESM.png]

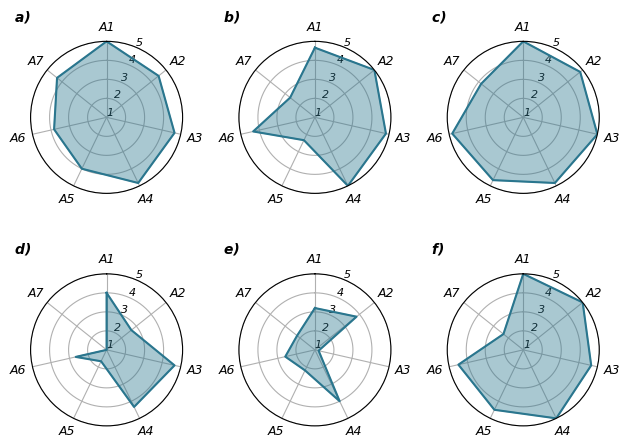

Supplement: Supplementary file 5 — Additional file 5: Figure S2. Radial Diagrams showing performance of the six highest performing tools in the user survey. The performance of the software tools in the user survey, considering the average score for each of the seven actions. The software tools in each plot are (a) Abstrackr, (b) Colandr, (c) Covidence, (d) DRAGON, (e) EPPI-Reviewer and (f) Rayyan. The actions are: (A1) creating an account, (A2) creating a systematic review project, (A3) importing references, (A4) inviting collaborators to join the project, (A5) carrying out T&Ab screening, (A6) exporting the screened references and (A7) finding and using the help section. [file 12874_2020_897_MOESM5_ESM.png]
